# Comfort and Person-Centered Care: Adaptation and Validation of the Colcaba-32 Scale in the Context of Emergency Services

**DOI:** 10.3390/nursrep15110383

**Published:** 2025-10-28

**Authors:** Maria do Céu Marques, Margarida Goes, Ana João, Henrique Oliveira, Cláudia Mendes, Rute Pires, Nuno Bravo

**Affiliations:** 1 Universidade de Évora, Comprehensive Health Research Centre (CHRC), Escola Superior de Enfermagem São João de Deus, Departamento de Enfermagem, 7000-811 Évora, Portugal; 2 Instituto Politécnico de Santarém, Escola Superior de Saúde, 2001-904 Santarém, Portugal; 3 Instituto de Telecomunicações, 3810-193 Aveiro, Portugal; 4 Escola Superior de Tecnologia e Gestão, Instituto Politécnico de Beja, 7800-295 Beja, Portugal; 5 Universidade de Évora, Comprehensive Health Research Center (CHRC), 7004-516 Évora, Portugal; 6 Departamento de Ciências e Tecnologias da Saúde, Escola Superior de Saúde/Instituto Politécnico de Portalegre, 7300-110 Portalegre, Portugal; 7 Unidade Local de Saúde Alto Alentejo, Serviço de Cirurgia, 7300-853 Portalegre, Portugal

**Keywords:** comfort, nursing, hospital emergency, instrument validation, psychometrics, Kolcaba

## Abstract

**Introduction:** Patient comfort is a central concept in nursing practice, and is particularly important in emergency contexts, where clinical complexity and care overload challenge the provision of humanized care. Katharine Kolcaba’s Theory of Comfort offers a robust theoretical framework for assessing and promoting comfort in multiple domains. The main objective is to psychometrically validate the adapted version of Kolcaba’s Comfort Scale—COLCABA-32—in critically ill patients treated in a Portuguese hospital emergency department. **Method:** A quantitative, descriptive, cross-sectional study was conducted using a sample of 165 adult patients triaged with urgent clinical priority. Data collection was performed through individual interviews. The COLCABA-32 Scale and the Mini-Mental State Examination (MMSE) were used. Statistical analysis included descriptive statistics, principal component analysis (PCA), internal consistency (Cronbach’s alpha), and correlation with clinical priority according to the Manchester Triage. **Results:** PCA revealed six factors with eigenvalues greater than 1, explaining 59.01% of the total variance of the scale. The dimensions identified were psycho-emotional comfort and autonomy, physical and symptomatic comfort, relational comfort and information, spiritual comfort, environmental comfort and motivational comfort and hope. The overall Cronbach’s alpha was 0.897, indicating excellent internal consistency. Correlations with clinical priority confirmed partial convergent validity. **Conclusions:** The COLCABA-32 Scale demonstrated adequate psychometric properties for assessing the comfort of critically ill patients in an emergency setting and is a valid, reliable, and sensitive instrument for the multiple dimensions of comfort, as proposed by Kolcaba. Its application can contribute to more person-centered and evidence-based nursing practices.

## 1. Introduction

Patient comfort is widely recognized as one of the fundamental pillars of nursing practice and has been valued since the time of Florence Nightingale, who advocated the influence of the physical and emotional environment on the recovery of the sick person [1]. Recent studies have also highlighted patient satisfaction as a care-sensitive outcome, particularly in older populations, reinforcing the need for valid instruments to measure subjective dimensions of care quality [2]. This evidence reinforces the research gap and the justification for adapting the COLCABA-32 to Portuguese emergency services, where the assessment of comfort remains underexplored despite its recognized clinical relevance.

Currently, Katharine Kolcaba’s Theory of Comfort offers a medium-range conceptual framework that allows comfort to be operationalized systematically and measurably. According to Kolcaba, comfort is a state in which the needs for relief, tranquility, and transcendence are satisfied in the physical, psychospiritual, sociocultural, and environmental domains [3,4,5]. This holistic approach, grounded in Kolcaba’s theoretical model, has been widely applied to evaluate comfort as a measurable and clinically operational construct [4,5,6,7]. This perspective is particularly relevant in complex clinical settings, such as emergency departments, where multiple factors can compromise the subjective experience of comfort [8,9,10,11].

In hospital emergency departments, healthcare professionals face significant challenges, such as time constraints, shortages of human and material resources, excessive workloads, and high patient turnover. These factors contribute to environments that are often impersonal, noisy, and depersonalized, making it difficult to provide individualized care and compromising the physical and emotional comfort of people in critical situations [12,13]. Systematic assessment of comfort has therefore been highlighted as essential to guide nursing interventions and to improve the therapeutic environment [14,15,16]. Evidence from rehabilitation nursing further supports the relevance of targeted interventions in complex clinical contexts, demonstrating improvements in self-care and functionality among older adults with respiratory disorders [17]. Several studies based on Kolcaba’s theory, conducted in different contexts of care for people in critical situations, indicate that these people often express feelings of pain, tiredness, insecurity, lack of privacy, and loss of autonomy [18,19,20,21]. The use of the instrument developed by the author allows the identification of care needs from the perspective of the sick person and is considered an indicator of the quality of nursing interventions [22,23].

In line with Kolcaba’s Theory of Comfort, comfort should be regarded as a measurable and clinically operationalizable phenomenon, rather than only a subjective feeling of well-being. Its systematic assessment allows for the identification of unmet needs and guides person-centered nursing interventions [24,25]. To this end, it is essential to have valid and culturally adapted instruments. One of the most recognized questionnaires is the General Comfort Questionnaire (GCQ), developed by Kolcaba, which is widely used in various contexts [26,27,28,29,30]. However, its application in emergency services, especially in Portugal, remains limited. The integration of patient-reported outcomes such as satisfaction, functionality, and quality of life into nursing care performance systems further underscores the need for culturally adapted and psychometrically validated instruments [7,24].

Although Kolcaba’s GCQ has been validated in different clinical and cultural contexts, there is still a clear gap concerning its psychometric validation for Portuguese emergency departments. In hospital emergency departments, care provision is constrained by multiple factors, such as excessive workloads, lack of time and human resources, often noisy and impersonal environments and high patient turnover. These factors compromise the care experience and can negatively affect the comfort of both critically ill patients and their families, who often report pain, fatigue, insecurity, lack of privacy and emotional distress [5].

Despite the growing relevance of the topic, the literature on comfort in emergency settings in Portugal remains limited and unsystematic. Although there are international studies that have validated comfort assessment tools in different clinical contexts, their use in Portuguese emergency services remains limited. Thus, a clear scientific gap persists: the lack of psychometrically validated and culturally adapted instruments that allow for the rigorous and systematic assessment of the comfort of people in critical situations treated in hospital emergency departments in Portugal [27].

To address this need, the present study aims to culturally adapt and psychometrically validate the COLCABA-32 Scale for the context of emergency departments in Portugal, assessing its validity and reliability properties. The designation COLCABA-32 results from the combination of “Comfort” and “Kolcaba”, thereby preserving the theoretical lineage of the original instrument. The application of this scale could constitute a sensitive and accurate diagnostic tool, allowing the identification of specific comfort needs and guiding more humanized, effective, and person-centered nursing practices.

## 2. Materials and Methods

### 2.1. Study Design

This was a quantitative, descriptive, and exploratory cross-sectional study aimed at psychometrically validating the adapted version of the Kolcaba Comfort Scale (General Comfort Questionnaire—32-item version), referred to in this study as the COLCABA-32 Scale. A methodological study was chosen to evaluate the measurement properties of the scale in a real clinical context, namely its construct validity, internal consistency, and semantic clarity, allowing for the systematic use of the scale in the context of critical patient care in emergency services in Portugal.

### 2.2. Instruments

The COLCABA-32 Scale is the result of the authorized adaptation of the GCQ, developed by Katharine Kolcaba (1992) [25], and has been adjusted to the context of emergency services in Portugal.

The cultural adaptation process followed the recommendations of the Consensus-based Standards for the Selection of Health Measurement Instruments (COSMIN) and was developed in several methodological stages [25]. In the first stage, the original version of the instrument was translated into European Portuguese by two independent translators, both with experience in the health field. The two versions were then compared and harmonized, resulting in a preliminary consensus version. Two bilingual translators, with no prior contact with the instrument, subsequently back translated it into English, ensuring semantic and conceptual equivalence with the original version. This version was then submitted for review by a committee of experts consisting of five specialists in nursing, research methodology, and linguistics, who assessed the cultural relevance, semantic clarity, and terminological adequacy of the items and proposed specific adjustments to better suit the Portuguese emergency context.

In the cultural adaptation of the Portuguese version of the COLCABA-32 scale, beyond the literal translation of the instrument, it was essential to incorporate the cultural specificities of the Portuguese context. This process involved not only linguistic translation but also a thorough analysis of the cultural differences that could affect the perception of comfort by Portuguese patients compared to the contexts where the scale was previously applied.

The spiritual dimension of comfort received special attention. Although spirituality is recognized as a relevant dimension in patient comfort across various cultures, in the Portuguese context, religious beliefs, especially Catholicism, play a significant role in patients’ responses. The adaptation aimed to reflect this cultural characteristic, ensuring that issues related to faith and spiritual beliefs were sensitive to local particularities, such as the value placed on spirituality as an important psychological and emotional resource for coping with crisis situations.

Additionally, when reviewing the cultural adaptation of the scale, it was noted that the environmental dimension could be further explored, as the physical environment of emergency units in Portugal can be a significant discomfort factor due to service overload and lack of privacy. The original scale did not adequately address the effects of factors such as constant noise and lack of privacy in Portuguese emergency services, elements that deeply affect the comfort experience of patients.

The differences observed during the adaptation process reflect the need for adjustments not only linguistically but also culturally, to ensure that the instrument is truly sensitive to the nuances of the Portuguese context. Thus, the adapted version of the COLCABA-32 takes these specificities into account while preserving the theoretical and structural essence of the original scale, which remains valid and relevant for the multidimensional assessment of comfort in emergency care.

In a subsequent stage, a cognitive pretest was conducted with a pilot group of patients (n = 30) to assess the comprehension of the items and the fluency of the language, with minor editorial changes being made based on the feedback obtained. Finally, the content validity index (CVI) was assessed by experts evaluating each item for relevance and clarity. The I-CVI (content validity index per item) and S-CVI (content validity index of the global scale) were calculated, obtaining values above 0.80 in all items, which ensures robust content validity.

The adaptation respected the original structure of the theory, maintaining the scope of the four domains of comfort (physical, psychospiritual, sociocultural, and environmental) and the three forms of comfort (relief, tranquility, and transcendence). Each item is answered on a 4-point Likert scale (1 = strongly disagree; 4 = strongly agree), with a higher score indicating greater perceived comfort. The total score ranges from 32 to 128 points. The Mini-Mental State Examination (MMSE) was also applied to ensure the cognitive integrity of the participants.

### 2.3. Target Population and Sample

The target population consisted of critically ill adult patients treated at the Emergency Department of the Local Health Unit. The inclusion criteria were age ≥ 18 years, preserved cognitive status (assessed by the MMSE score ≥ 24 points), verbal communication ability, and free and informed consent. Patients with cognitive impairment, clinical instability or under sedation were excluded.

Concerning clinical priority, patients triaged according to the Manchester Triage System at orange, yellow, and green levels were included to reflect the reality of the population treated. The inclusion of green priority patients was assumed to allow for greater clinical variability in the sample and to ensure the robustness of the psychometric analysis.

The sample included 165 participants, a number considered adequate for principal component analysis and internal consistency, complying with the recommendation of at least 5 to 10 participants per scale item [26]. The sex distribution revealed a predominance of females (59.4%) over males (40.6%) (Table 1).

In terms of age, patients ranged from 18 to 88 years old, with a mean age of 53.7 years and a standard deviation of 18.67 years, reflecting a predominantly adult and elderly population. The age distribution is wide, with a peak between 60 and 70 years, with 62 being the most common age group (6.7%) (Table 1).

With respect to education, most patients had a high school education (35.8%), followed by elementary school (34.5%) and basic education (26.1%). Only a small fraction had higher education—3% with a bachelor’s degree and 0.6% with a master’s degree, indicating a predominantly low educational level (Table 1).

Almost all individuals (97.6%) had previously used the emergency service, suggesting a pattern of continuous use of this type of care.

The Manchester Triage Scale, used to classify the severity of cases, revealed a relatively balanced distribution among the three levels present: yellow (35.2%), green (33.3%), and orange (31.5%), indicating that most patients presented with moderate severity (Table 1).

### 2.4. Application Procedures

Data collection was carried out through face-to-face interviews conducted in the emergency department. To ensure the reliability of the collection, interviewers received standardized prior training, including simulation sessions and joint review of the instrument application script.

The interviews lasted an average of 10 to 15 min per participant. Whenever possible, they were conducted in spaces that ensured privacy, with an effort to minimize interference from the typical noise of the emergency room environment.

To reduce social desirability bias, interviewers were instructed to adopt a neutral stance, explaining in advance to participants that there were no right or wrong answers and that the data would be treated anonymously and confidentially.

### 2.5. Statistical Treatment

Data analysis was performed using SPSS software (version 29). Descriptive statistics, namely frequencies, means, and standard deviations, were applied to characterize the sample and the scale items. The psychometric validation of the COLCABA-32 involved different procedures. Internal consistency was assessed using Cronbach’s alpha coefficient, considering values equal to or greater than 0.70 to be acceptable, and complemented by Spearman–Brown’s coefficient analysis, which allows the stability of the measure to be verified. The latent structure of the scale was examined using Principal Component Analysis (PCA), the adequacy of which was previously confirmed using the Kaiser–Meyer–Olkin (KMO) test and Bartlett’s sphericity test. The factors were extracted using the principal component method with varimax orthogonal rotation, retaining factors with eigenvalues greater than 1 and considering items with factor loadings equal to or greater than 0.40 to be valid. Additionally, the corrected item–total correlation was calculated to assess the internal homogeneity of the items, ensuring the consistency of the scale.

### 2.6. Ethical Considerations

The study design, methods used, data collection procedures, and the free and informed consent form presented to each participant were approved by the following Ethics Committees: (i) Ethics Committee of the Polytechnic Institute of Portalegre and (ii) Ethics Committee of the Local Health Unit of Alto Alentejo. The study is registered under protocol number 39. All ethical procedures fully complied with the principles of the Declaration of Helsinki, safeguarding the dignity, privacy, and freedom of the participants. In addition, the author of the original scale, Katharine Kolcaba, granted formal authorization by email for the adaptation and use of the scale in this study.

## 3. Results

### 3.1. Suitability of the Sample for Factor Analysis

The study of the psychometric properties of COLCABA-32 was conducted with a sample of 165 participants. The suitability for factor analysis was assessed using the KMO (Kaiser–Meyer–Olkin Measure of Sampling Adequacy) index and Bartlett’s sphericity test. Marôco (2010) explained that the KMO index is a measure of the homogeneity of variables, which compares simple correlations with partial correlations between variables [27]. According to the same author, the values obtained in this test follow the recommendations presented in the table below (Table 2).

In this study, the adequacy of the application of factor analysis was evaluated using the KMO index, which has value was 0.879 and is considered excellent. Bartlett’s sphericity test presented a value of χ^2^ (496) = 2716.64, with statistical significance (*p* < 0.001) indicating a significant correlation between the variables (Table 2). Thus, based on these results, it can be stated that, with the present sample, the scale is suitable for factor analysis.

### 3.2. Principal Component Analysis

Principal Component Analysis was conducted on the COLCABA-32 Scale (Table 3), identifying 6 factors with eigenvalues greater than 1, in line with Kaiser’s criterion. This factor solution accounted for 59.01% of the total variance, which is considered adequate for multidimensional instruments applied in complex clinical settings. However, it is important to note that the structure obtained did not fully replicate the factor solution proposed by the original scale’s author, likely reflecting cultural and contextual specificities of the studied population.

While the explained variance is deemed acceptable for instruments with multiple dimensions in complex clinical contexts, we recognize that it may be seen as relatively low when compared to other analyses. This lower percentage highlights the complexity of the comfort concept, which encompasses multiple interdependent factors, with each dimension of comfort not being fully captured by a single factor.

The six factors identified, with eigenvalues greater than 1, explained 59.01% of the total variance. The first factor corresponded to 31.704% of the total variance of the instrument, with an eigenvalue of 10.15, and consisted of eight items. The second factor represented 8.63% of the total variance, with an eigenvalue of 2.76, and consisted of eight items. The third factor, with 7 items, explained 7.34% of the variance and had an eigenvalue of 2.35.

The fourth factor, consisting of 2 items, had an eigenvalue of 1.66 and explained 5.20% of the total variance. The fifth factor, with 2 items and an eigenvalue of 1.28, explained 4.0% of the variance. Finally, the sixth factor, with only 2 items and an eigenvalue of 1.20, explained 3.76% of the total variance (Table 2).

**Figure 1:**
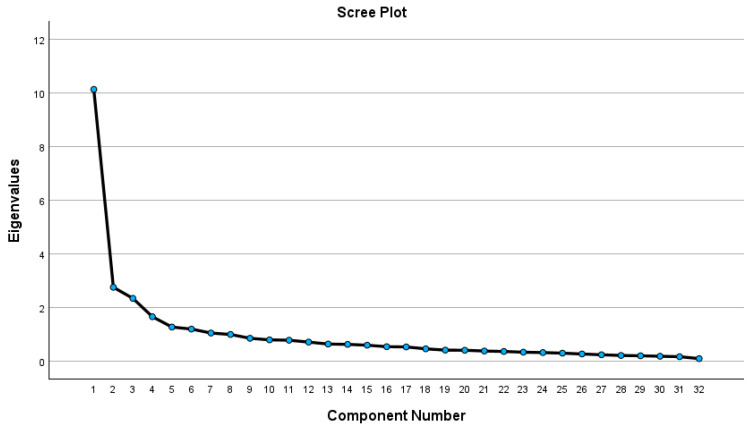
Scree plot graph of the factorization of the Colcaba-32.

The scree plot also indicated the presence of 5 to 6 factors, and it was decided to use 6 factors, a value obtained through principal component analysis (Figure 1).

### 3.3. Identified Factor Structure

For the factor analysis, the varimax orthogonal rotation method was used, and six factors were retained as basis of the Kaiser criterion (eigenvalues greater than 1). To ensure the quality of the factor solution, only items with factor loadings equal to or greater than 0.40 were considered, as recommended by Hair et al. (2014) and Marôco (2010) [26,27,28]. In this process, items 9, 14, 16, 21, 25, 30, and 31 were excluded because they had factor loadings below the established threshold, indicating low representativeness in the identified structure. Additionally, items 2, 17, and 25 were removed because they were not consistently associated with any of the extracted factors and thus did not contribute to the robustness and consistency of the factor solution (Table 4).

The analysis of commonalities shows that most items on the scale have satisfactory values, ranging from 0.55 to 0.88, which indicates that a substantial proportion of the variance of each item is explained by the extracted factors (Table 4).

The highest values were observed in the items of the Spiritual Comfort dimension, namely *“My faith helps me not to be afraid”* (h^2^ = 0.863) and *“My beliefs give me peace of mind”* (h^2^ = 0.883), which reveals a strong contribution of these items to the factorial structure. High commonalities also stand out in items associated with physical and psycho-emotional discomfort, such as *“I feel out of control”* (h^2^ = 0.691), *“I feel uncomfortable because I am not dressed”* (h^2^ = 0.663), *“I do not feel healthy at the moment”* (h^2^ = 0.726), *“My health makes me sad”* (h^2^ = 0.723), and *“I am afraid of what is going to happen”* (h^2^ = 0.740) (Table 4).

On the other hand, some items had lower, albeit still acceptable, communalities, such as *“No one understands me”* (h^2^ = 0.552), *“I am very tired”* (h^2^ = 0.609), and *“I don’t like this place”* (h^2^ = 0.577). These values suggest that these items, despite being associated with the overall construct, explain a smaller proportion of its variance (Table 4).

Overall, the results indicate that most items have commonalities above the minimum recommended value of 0.40, confirming the appropriateness of their inclusion in the factorial model and supporting the structural validity of the scale.

Based on the factor analysis performed on the COLCABA-32 Scale, and in light of Katharine Kolcaba’s Theory of Comfort, it was possible to identify six distinct dimensions that reflect different domains and forms of comfort experienced by critically ill patients in an emergency department setting. The names given to these dimensions sought to respect the conceptual structure proposed by Kolcaba [24], namely the four domains of comfort (physical, psychospiritual, sociocultural, and environmental) and the three forms it can take (relief, tranquility, and transcendence).

The first dimension was named Psycho-emotional Comfort and autonomy, as it groups items that express feelings of dependence, loss of control, incomprehension, and lack of belonging, in contrast to self-confidence. This dimension refers to the psycho-spiritual domain, as it reflects how patients interpret and address their condition and identity in the disease process, influencing their tranquility and personal integrity.

The second dimension, Physical and Symptomatic Comfort, includes items related to the relief of symptoms such as pain, fatigue, bodily discomfort, and the need for rest, as well as aspects of the immediate environment that directly affect physical well-being. This dimension is clearly rooted in the physical domain of Kolcaba’s theory, corresponding to the form of comfort referred to as relief.

The third dimension was entitled Relational Comfort and Information, which consists of items that highlight the importance of contact with the healthcare team and clear information about the clinical condition. The satisfaction of these needs contributes to patients’ peace of mind and security, reflecting the sociocultural domain of comfort, particularly with regard to interpersonal interaction and communication.

The fourth dimension, Spiritual Comfort, concerns patients’ spiritual experience and is represented by items that address faith and personal beliefs as sources of serenity and security in the face of illness. This dimension is closely linked to the psychospiritual domain and to the form of comfort called transcendence, defined by Kolcaba as the ability to face adversity with meaning and inner elevation.

The fifth dimension, called Environmental Comfort, covers the patient’s perception of the physical space in which they find themselves, including thermal comfort and the familiarity or strangeness of the place. This dimension falls within the environmental domain, which includes all external elements that influence a person’s comfort.

Finally, the sixth dimension, called Motivational Comfort and Hope, includes items that express the need for recovery, future well-being, and the influence of the mood of the environment on the patient’s emotional state. This dimension corresponds to the form of transcendental comfort, in that it represents the overcoming of present suffering through the projection of a more favorable future.

### 3.4. Descriptive Statistics of the Dimensions

The descriptive analysis of the six dimensions of the COLCABA-32 Scale allowed us to characterize the levels of comfort perceived by the participants in the different areas evaluated (Table 5).

The psycho-emotional comfort and autonomy dimension had the highest mean score (M = 2.94; SD = 0.58), indicating that participants tended to report relatively high levels of comfort related to self-confidence, interpersonal understanding and perceived control over their situation (Table 5).

The Spiritual Comfort dimension had a similar average (M = 2.91; SD = 0.66), suggesting that personal beliefs and spirituality are often cited as sources of support and tranquility in emergency contexts (Table 5).

On the other hand, the Relational Comfort and Information (M = 1.97; SD = 0.61) and Motivational Comfort and Hope (M = 1.98; SD = 0.35) recorded lower averages, revealing possible gaps in communication between the team and the user, as well as in the ability of patients to positively project the near future (Table 5).

The physical and Symptomatic Comfort dimension had a moderate mean (M = 2.16; SD = 0.67), reflecting the presence of uncomfortable symptoms such as pain and fatigue, but also some preservation of physical comfort in part of the sample (Table 5).

Finally, the Environmental Comfort dimension had a mean of 2.28 (SD = 0.52), which shows a reasonable perception of the conditions of the physical environment, such as temperature and privacy, although there is still room for improvement (Table 5).

### 3.5. Internal Consistency

The internal consistency of the COLCABA-32 Scale was assessed using Cronbach’s alpha coefficient, both for the total scale and for each of the six dimensions identified in the principal component analysis. In psychometric analysis, the item–total correlation reflects how well each question (item) relates to the overall scale score. In simple terms, it shows whether an item is consistent with the rest of the instrument—that is, whether people who score high on the total scale also tend to score high on that particular question. Higher correlations indicate that the item measures the same concept as the rest of the scale.

The overall Cronbach’s alpha value was 0.897 and the Spearman–Brown coefficient was 0.892, indicating high internal reliability and confirming the homogeneity of the items that make up the scale (Table 6).

The analysis of the internal consistency of the COLCABA-32 Scale revealed generally satisfactory values, demonstrating that the different factors have adequate levels of reliability. The psycho-emotional comfort and autonomy dimension had a Cronbach’s alpha of 0.865, with item–total correlations ranging from 0.492 to 0.715, and the Spearman–Brown coefficient ranged from 0.712 to 0.832, confirming the stability of the measure. The Physical and Symptomatic Comfort dimension stood out with a Cronbach’s alpha of 0.887 and item–total correlations between 0.599 and 0.725, with the Spearman–Brown coefficients ranging from 0.873 to 0.875, which reinforces the high consistency of this dimension. The Relational and Information Comfort dimension also presented acceptable results, with a Cronbach’s alpha of 0.703 and item–total correlations of 0.542, a value also obtained for the Spearman–Brown coefficient, which demonstrates adequate consistency despite the small number of items (Table 6).

The Spiritual Comfort dimension had the highest internal consistency value, with a Cronbach’s alpha of 0.914 and an item–total correlation of 0.842, confirming the homogeneity of the items that constitute it, a result reinforced by the Spearman–Brown coefficient, which was also 0.914. In contrast, the Environmental Comfort dimension recorded the lowest value, with a Cronbach’s alpha of 0.600 and item–total correlations of 0.384, placing it at the lower threshold of acceptability; this result may be related to the small number of items and the lower variability of responses, so it should be interpreted with caution and reevaluated in future studies. The Motivational Comfort and Hope dimension obtained a Cronbach’s alpha of 0.758, with relatively low item–total correlations (0.284), a value confirmed by the Spearman–Brown coefficient (0.759), revealing acceptable internal consistency, although there was room for improvement (Table 6).

Overall, the results show that the COLCABA-32 Scale has good internal consistency and stability, especially in the dimensions related to Psycho-emotional, physical, and Spiritual Comfort, which had the highest reliability values. The motivational/hope and relational/information dimensions recorded acceptable levels of internal consistency, whereas the environmental dimension presented more modest values, but they were still within the minimum limits recommended in the literature [28]. The analysis of the corrected item–total correlations revealed, for the most part, coefficients above 0.40, indicating a positive contribution of the items to the internal consistency of the scale. Items with lower values, although above the recommended minimum limit (≥0.30), were retained because they were theoretically relevant to the conceptual domain of comfort (Table 6).

These results confirm the psychometric soundness of the adapted version of the scale, demonstrating that the COLCABA-32 is a reliable instrument for assessing different dimensions of comfort in critically ill patients in the hospital emergency setting.

### 3.6. Correlations Between the Dimensions of the COLCABA-32 Scale and the Structural Consistency of the Instrument

Spearman’s correlation analysis between the dimensions of the COLCABA-32 Scale revealed statistically significant associations between most subscales, demonstrating a coherent internal structure aligned with the theoretical proposal of the scale (Table 7).

The highest correlations with the total scale score were observed in the Physical and Symptomatic Comfort dimension (r = 0.916; *p* < 0.001) and the Psycho-emotional Comfort and Autonomy dimension (r = 0.813; *p* < 0.001), indicating that these dimensions hold significant weight in the participants’ overall perception of comfort. Positive correlations were also found with the Motivational Comfort and Hope dimension (r = 0.569; *p* < 0.001), Environmental Comfort (r = 0.564; *p* < 0.001), and Relational Comfort and Information (r = 0.425; *p* < 0.001), further reinforcing the multidimensional nature of the comfort construct (Table 7).

Notably, the correlation between the Psycho-emotional Comfort and Autonomy and Physical and Symptomatic Comfort dimensions was moderately strong (r = 0.651; *p* < 0.001), suggesting a close interrelation between the patient’s emotional experience and their physical state. Additionally, moderate correlations were observed between Physical and Environmental Comfort (r = 0.486; *p* < 0.001), Physical and Motivational Comfort (r = 0.489; *p* < 0.001), Psycho-emotional and Motivational Comfort (r = 0.396; *p* < 0.001), and Relational and Environmental Comfort (r = 0.356; *p* < 0.001). These results indicate that both physical and emotional well-being simultaneously influence the perceived environment and the patient’s hope about their health status (Table 7).

Although the correlations between some dimensions were of lower magnitude, they remained statistically significant. For example, the correlation between Relational Comfort and Information and Physical and Symptomatic Comfort (r = 0.345; *p* < 0.001) and between Relational Comfort and Motivational Comfort (r = 0.173; *p* = 0.026) highlight the importance of interpersonal connections and communication with healthcare providers in fostering comfort (Table 7).

In contrast, the Spiritual Comfort dimension did not show significant correlations with the total scale score (r = −0.125; *p* = 0.110) nor with most of the other dimensions, except for weak negative correlations with Psycho-emotional Comfort (r = −0.221; *p* = 0.004) and Physical Comfort (r = −0.202; *p* = 0.009). These findings underscore the distinctiveness of the spiritual dimension, suggesting that it functions as an autonomous aspect of comfort, minimally influenced by the physical or emotional domains. This is indicative of the divergent validity of the instrument (Table 7).

Overall, the data support the conceptual framework of the scale and confirm the internal consistency of its subdimensions, showing clear convergence between the more interdependent factors and relative autonomy among the more subjective dimensions, such as Spiritual Comfort.

### 3.7. Convergent and Divergent Validity

Spearman’s correlation analysis between the six dimensions of the COLCABA-32 Scale and the Manchester Triage classification allowed us to explore the convergent validity of the instrument, particularly concerning the relationship between perceived comfort and the clinical severity of patients in an emergency context (Table 8).

The Physical and Symptomatic Comfort dimension showed the highest correlation with clinical priority (r = 0.740; *p* < 0.001), suggesting that patients classified as more severe experience lower levels of physical comfort. This result empirically validates the clinical relevance of this dimension, since symptoms such as pain, bodily discomfort, and fatigue tend to be more intense in acute clinical situations, as predicted by Kolcaba’s Theory of Comfort [24] (Table 8).

The Psycho-emotional Comfort and Autonomy dimension also revealed a significant correlation (r = 0.452; *p* < 0.001), indicating that as clinical severity increases, patients feel less autonomous and more emotionally vulnerable. This association reinforces the importance of active listening and promoting a sense of control and security as fundamental strategies for mitigating psycho-emotional distress in critical contexts (Table 8).

The dimensions Motivational Comfort and Hope (r = 0.318; *p* < 0.001) and Environmental Comfort (r = 0.309; *p* < 0.001) also showed significant correlations, albeit of lesser magnitude. These findings suggest that even in more severe clinical states, aspects such as hope for recovery and the impact of the physical environment (temperature, noise and familiarity of the space) remain relevant to the experience of comfort. These results are consistent with the holistic approach proposed by Kolcaba, according to which comfort transcends physical relief, also integrating motivational and environmental dimensions (Table 8).

In contrast, the dimensions of Relational Comfort and Information (r = 0.140; p = 0.074) and Spiritual Comfort (r = −0.124; *p* = 0.113) did not show statistically significant correlations with clinical priority. This lack of association can be interpreted as an indication that these forms of comfort are less influenced by the immediate clinical condition and more dependent on personal, relational, and contextual factors. The spiritual dimension seems to remain a stable internal resource, not necessarily conditioned by the severity of the health condition, which gives the scale discriminant validity (Table 8).

These results reinforce the validity of the COLCABA-32 Scale, showing that the dimensions with the highest symptomatic or contextual load are directly associated with clinical severity, while other dimensions—more subjective or existential—reveal relative independence. Thus, the scale’s ability to discriminate between and evaluate different dimensions of comfort is confirmed, supporting its application in emergency contexts as a sensitive, multidimensional, and theoretically robust instrument.

The results also suggest evidence of divergent validity, since the Spiritual and Relational dimensions did not show a significant correlation with clinical priority, indicating that these forms of comfort are not directly associated with the severity of the health condition, but rather with subjective and relational factors. This finding reinforces the multidimensionality of comfort, as proposed by Kolcaba’s Theory.

## 4. Discussion

The psychometric validation of the adapted version of Kolcaba’s Comfort Scale (COLCABA-32) identified a structure composed of six factors, which represent different dimensions of the comfort experience of critically ill patients in an emergency hospital setting. This structure confirms the multidimensional nature of comfort in line with the definition proposed by Kolcaba (2003) [24], who conceptualizes it as a holistic state experienced in multiple domains—physical, psychospiritual, sociocultural, and environmental—and expressed in the forms of relief, tranquility and transcendence. Rather than merely reproducing the factor solution, these findings highlight the complexity of the comfort construct when applied to critically ill patients in emergency settings, where vulnerability, unpredictability, and contextual constraints shape the perception of comfort.

The principal component factor analysis showed excellent data adequacy, with a KMO value of 0.879 and a statistically significant Bartlett’s sphericity test (*p* < 0.001), indicating a good correlation between items and justifying the application of the technique. The six factors extracted explain about 59% of the total variance of the scale. Although this percentage falls below some thresholds reported in the literature, it remains acceptable given the heterogeneity of critically ill patients and the complexity of the construct [27,28]. Importantly, moderate correlations observed between some factors (e.g., r = 0.356) may be statistically significant but should be interpreted as partial, rather than strong, overlaps. Clinically, this suggests that each dimension captures distinct yet complementary aspects of comfort, justifying the multidimensional approach.

The Psycho-emotional Comfort and Autonomy dimension proved to be particularly relevant, integrating items associated with the perception of control, independence, belonging, and understanding, which are fundamental aspects for maintaining the dignity and identity of patients in critical situations. This dimension highlights the importance of autonomy and active listening as essential components of comfort, especially in contexts such as the emergency department, where the experience of vulnerability is particularly acute [11,18]. These findings resonate with previous studies emphasizing that preserving dignity and identity is critical in settings where loss of autonomy is common.

The second dimension, Physical and Symptomatic Comfort, covered aspects related to pain relief, rest, and privacy, confirming that the physical domain remains central to the assessment of comfort. However, the simultaneous presence of items related to safety and fear shows that physical symptoms cannot be dissociated from emotional experience, reinforcing the interdependence between the domains of comfort [24]. This supports Kolcaba’s proposition that comfort must be assessed holistically rather than in isolation.

The Relational Comfort and Information dimension reinforces the importance of interpersonal relationships and effective communication in building comfort, corroborating previous studies that highlight the need for clear information and emotional support as determinants of patient tranquility [9,10,13,14]. The absence of information or its inaccessibility can accentuate suffering and insecurity, becoming an obstacle to the humanization of care. This dimension demonstrates that clarity of information and emotional presence are not ancillary but fundamental determinants of tranquility in critical contexts.

The Spiritual Comfort dimension, although composed of only two items, showed adequate internal consistency and clinical relevance. Faith and religious beliefs are coping resources that can mitigate uncertainty and suffering, promote transcendence and contributing to psychological and emotional homeostasis. This result is in line with the assumptions of Comfort Theory, which recognizes spirituality as a modulating factor in the experience of illness and the perception of well-being [24].

However, the absence of significant correlations with the total comfort scale, alongside the presence of weak negative correlations with physical and psycho-emotional comfort, suggests that spirituality may operate as an autonomous construct. This finding could reflect cultural particularities in Portugal, where spirituality is often perceived as a private and individualized domain rather than a universal health resource. In the immediacy of emergency care, spirituality may thus emerge as an idiosyncratic coping mechanism, relatively independent of other comfort dimensions.

The lack of significant correlations between Spiritual Comfort and other domains may also point to specific cultural and contextual factors. In Portugal, spirituality is frequently intertwined with religious beliefs—predominantly Catholic—that are regarded as personal and internal resources, rather than as dimensions openly integrated into healthcare practice. In emergency settings, where the primary focus lies on immediate physical stabilization, manifestations of faith or spiritual needs may remain underacknowledged by professionals and privately experienced by patients. This cultural dynamic may help explain why the spiritual dimension functions as an autonomous aspect of comfort, relatively distinct from the physical or emotional domains.

The identification of the Environmental Comfort dimension confirms that factors such as temperature, noise, and the overall physical environment have a direct impact on patient well-being, particularly in contexts such as emergency services, which are often overcrowded and depersonalized. These results corroborate the principles advocated, with the recognized importance of the environment in patient recovery [29]. They also reinforce the value of considering environmental factors as modifiable elements in the pursuit of more humanized emergency care.

Finally, the Motivational Comfort and Hope dimension shows that mood, the projection of improvement, and the desire for recovery are essential subjective elements for facing illness with optimism. This dimension translates into a form of transcendence, in which comfort is not restricted to the absence of suffering, but also includes the ability to give meaning to the experience of illness and to visualize a possible future [14,15,16,18]. These aspects illustrate that comfort is both restorative and motivational, enabling patients to reframe their illness experience.

The six-factor structure now identified not only reflects the theoretical assumptions of Comfort Theory but also introduces a contextualized reading of the reality of Portuguese emergency services. The moderate inter-factor correlations suggest that comfort is multidimensional and partially independent across domains, supporting the use of a multidimensional scale rather than a unidimensional score.

Additionally, the Cronbach’s alpha coefficients obtained for the different factors ranged from 0.60 to 0.91, values generally considered acceptable to high in social sciences [30]. These results give statistical robustness to the COLCABA-32 version and support its applicability as a tool for the systematic assessment of comfort in emergency services. Nevertheless, the spiritual dimension, given its limited number of items and lower internal consistency, may benefit from further refinement or expansion. Future studies should also conduct confirmatory factor analysis (CFA) to test the stability of the six-factor structure in independent samples.

## 5. Limitations

Despite the encouraging results, this study has some limitations that should be acknowledged. First, the use of a convenience sample, collected from a single hospital emergency department, limits the generalizability of the results to other contexts and populations. In addition, the sample did not include patients admitted to intensive care units or those who were unable to communicate, which restricts the applicability of the findings to more severe or non-communicative populations. There was no independent confirmatory factor analysis (CFA), which could have reinforced the structural validity of the instrument. Some dimensions of the scale were represented by only two items, which may limit its factorial stability. This is particularly relevant for the Spiritual Comfort dimension, which showed lower internal consistency and may require refinement or expansion.

Given these limitations, future studies should seek to validate the factorial structure of COLCABA-32 through confirmatory analyses in independent samples, as well as test its temporal stability through test–retest studies. Assessing criterion validity, particularly through its association with relevant clinical indicators such as pain, anxiety, or environmental noise, is another important step. Cross-cultural validation in different hospital and emergency contexts will also be necessary to extend the applicability of the instrument beyond a single Portuguese setting. Finally, it is suggested that sensitivity to change be analyzed to verify whether the scale can capture variations in comfort in response to specific nursing interventions or changes in the clinical environment.

## 6. Conclusions

This research allowed for the psychometric validation of the COLCABA-32 Scale, an adapted and contextualized version of Katharine Kolcaba’s General Comfort Questionnaire aimed at assessing comfort in critically ill patients in an emergency hospital setting. The results obtained demonstrated that the scale has good psychometric properties, particularly in terms of construct validity and internal consistency, with a high overall Cronbach’s alpha (α = 0.897) and a factorial structure composed of six dimensions consistent with the domains and forms of comfort defined by Kolcaba’s Theory.

PCA identified six distinct factors: Psycho-emotional Comfort and Autonomy, Physical and Symptomatic Comfort, Relational Comfort and Information, Spiritual Comfort, Environmental Comfort, and Motivational Comfort and Hope, which together explained 59% of the total variance of the scale. These factors reflect the complexity and multidimensionality of the comfort experience in critical situations, reinforcing the theoretical relevance of the holistic approach proposed by Kolcaba.

The correlations between the dimensions of the scale and clinical severity, as measured by the Manchester Triage, showed convergent validity in the dimensions most associated with symptoms and physical vulnerability. At the same time, the spiritual and relational dimensions did not show significant correlations with clinical priority, suggesting divergent validity and reinforcing the specificity of these domains as autonomous components of the comfort experience.

In this sense, the COLCABA-32 Scale proves to be a valid, reliable and sensitive instrument, capable of capturing different dimensions of comfort in demanding clinical contexts. Its clinical applicability lies in supporting diagnostic assessment, informing individualized care planning, and promoting the humanization of nursing practice in emergency departments. By identifying unmet comfort needs, the scale may contribute to more accurate, person-centered interventions and to the continuous monitoring of care quality.

We suggest conducting future research using the scale to develop more targeted and evidence-based nursing interventions, as well as to explore the correlation between comfort and other clinical variables, such as pain or anxiety. It will also be important to validate its structure through confirmatory factor analysis (CFA) in independent samples, refine the Spiritual Comfort dimension, and extend its use to populations not included in this study, such as non-communicative patients and those admitted to intensive care units. Additionally, it will be pertinent to validate its application in specific populations, such as pediatrics or geriatrics, and to conduct longitudinal studies to understand the evolution of comfort throughout the patient’s journey in the emergency department. In this way, the COLCABA-32 can evolve from being only a measurement tool to becoming a catalyst for innovation and continuous improvement in the quality and humanization of nursing care.

## Figures and Tables

**Table 1:** Demographic and Clinical Characterization of the Population Treated in the Emergency Department (n = 165).

Variable	Category	Frequency	%
Gender	Male	67	40.6
	Female	98	59.4
Age groups	18–29	25	15.2
	30–39	25	15.2
	40–49	22	13.3
	50–59	31	18.8
	60–69	38	23
	70–79	25	15.2
	80–89 years old	9	5.5
Education	Primary	57	34.5
	Basic education	43	26.1
	Secondary Education	59	35.8
	Bachelor’s Degree	5	3.0
	Master’s degree	1	0.6
First time in the emergency room?	Yes	4	2.4
	No	161	97.6
Manchester Triage	Orange	52	31.5
	Yellow	58	35.2
	Green	55	33.3

**Table 2:** KMO and Bartlett’s test for the Colcaba-32.

KMO Measure (Kaiser–Meyer–Olkin of Sample Adequacy)		0.879
Bartlett’s test (Sphericity test)	Chi-square	2716.646
	Df	496
	Sig.	<0.001

**Table 3:** Eigenvalues and variance explained by factor.

Items	Eigenvalue	% Variance	% Variance Accumulated	Amount Own	% Variance	% Variance Accumulated
1	10.145	31.704	31.704	4.999	15.621	15.621
2	2.763	8.634	40.338	4.887	15.272	30.893
3	2.349	7.342	47.680	2.673	8.352	39.245
4	1.664	5.199	52.880	2.401	7.504	46.749
5	1.279	3.995	56.875	2.338	7.305	54.054
6	1.203	3.758	60.633	1.586	4.956	59.010

**Table 4:** Factor matrix with loadings greater than 0.40 (Varimax rotation).

Items	Factors						h^2^
	1	2	3	4	5	6	
Psycho-emotional and Autonomy							
28—I feel out of control (reversed)	0.761						0.691
P29—I feel uncomfortable because I am not dressed (reversed)	0.759						0.663
P5—I feel dependent on others (Reversed)	0.724						0.713
P24—My belongings are not here (Reversed)	0.649						0.742
P12—I am constipated at the moment (Reversed)	0.637						0.638
P7—No one understands me (Reversed)	0.625						0.552
P4—I feel confident.	0.589						0.582
P20—I am very tired (Reversed)	0.520						0.609
Physical and Symptomatic							
P13—I don’t feel healthy right now (Reversed)		0.757					0.726
P3—My health makes me sad (Reversed)		0.755					0.723
P6—Noise prevents me from resting (Reversed)		0.687					0.632
P8—My pain is difficult to bear (Reversed)		0.638					0.700
P23—This chair/bed hurts me (Reversed)		0.625					0.687
P1—I have enough privacy		0.588					0.689
P15—I am afraid of what is about to happen (Reversed)		0.544					0.740
Relational and Information							
P27—I need to be better informed about my health status (Reversed)			0.791				0.648
P18—I would like to see my doctor more often (Reversed)			0.783				0.712
Spiritual							
P10—My faith helps me not to be afraid				0.908			0.863
P26—My beliefs give me peace of mind				0.906			0.883
Environmental							
P11—I don’t like this place (Reversed)					0.737		0.577
P19—The temperature in this place is pleasant					0.649		0.673
Motivational and Hope							
P32—I need to feel good again (Reversed)						0.741	0.698
P22—The mood here makes me feel better						0.514	0.665

**Table 5:** Descriptive statistics of the dimensions of the COLCABA-32 Scale.

COLCABA-32 Dimension	Mean	Deviation	Minimum	Maximum
Psycho-emotional and Autonomy	2.94	0.58	1.63	4.0
Physical and Symptomatic	2.16	0.67	1.00	3.86
Relational and Information	1.97	0.61	1.00	4.0
Spiritual	2.91	0.66	1.00	4.00
Environmental	2.28	0.52	1.00	4.00
Motivation and Hope	1.98	0.35	1.00	2.50

**Table 6:** Internal consistency and stability of the COLCABA-32 Scale: Cronbach’s alpha coefficients, Spearman–Brown coefficients, and item–total correlations by dimension.

Factors	Items	Item–total Correlation
Factor I—Psycho-emotional Comfort and Autonomy Cronbach’s alpha = 0.865 Spearman–Brown = 0.712/0.832	28	0.702
	29	0.690
	5	0.715
	24	0.559
	12	0.492
	7	0.574
	4	0.612
	20	0.598
Factor II—Physical and Symptomatic Comfort Cronbach’s alpha = 0.887 Spearman–Brown = 0.873/0.875	13	0.725
	3	0.599
	6	0.683
	8	0.707
	23	0.694
	1	0.633
	15	0.722
Factor III—Relational Comfort and Information Cronbach’s alpha = 0.703 Spearman–Brown = 0.703	27	0.542
	18	0.542
Factor IV—Spiritual Comfort Cronbach’s alpha = 0.914 Spearman–Brown = 0.914	10	0.842
	26	0.842
Factor V—Environmental Comfort Cronbach’s alpha = 0.600 Spearman–Brown = 0.600	11	0.384
	19	0.384
Factor VI—Motivational Comfort and Hope Cronbach’s alpha = 0.758 Spearman–Brown = 0.759	32	0.284
	22	0.284

**Table 7:** Correlations between the dimensions of the COLCABA-32 Scale and total scale.

		Psycho-emotional and Autonomy	Physical and Symptomatic	Relational Information	Spiritual	Environmental	Motivational and Hope
Colcaba Total Scale	r	0.813 **	0.916 **	0.425 **	−0.125	0.564 **	0.569 **
	*p*	<0.001	<0.001	<0.001	0.110	<0.001	<0.001
Psycho-emotional and Autonomy	r	1.000	0.651 **	0.181 *	−0.221 **	0.261 **	0.396 **
	*p*	-----	<0.001	0.020	0.004	<0.001	<0.001
Physical and Symptomatic	r	0.651 **	1.000	0.345 **	−0.202 **	0.486 **	0.489 **
	*p*	<0.001	-----	<0.001	0.009	<0.001	<0.001
Relational and Information	r	0.181 *	0.345 **	1.000	−0.134	0.356 **	0.173 *
	*p*	0.020	<0.001	-----	0.086	<0.001	0.026
Spiritual	r	−0.221 **	−0.202 **	−0.134	1.000	0.053	−0.074
	*p*	0.004	0.009	0.086	-----	0.497	0.342
Environmental	r	0.261 **	0.486 **	0.356 **	0.053	1.000	0.454 **
	*p*	<0.001	<0.001	<0.001	0.497	-----	<0.001
Motivational and Hope	r	0.396 **	0.489 **	0.173 *	−0.074	0.454 **	1.000
	*p*	<0.001	0.916 **	0.026	0.342	<0.001	-----

Legend: * *p* < 0.05, ** *p* < 0.01.

**Table 8:** Spearman’s correlation between comfort and clinical priority.

COLCABA-32 Dimensions		Manchester Triage
Psycho-emotional comfort and autonomy	r	0.452 **
	*p*	<0.001
Physical and Symptomatic Comfort	r	0.740 **
	*p*	<0.001
Relational Comfort and Information	r	0.140
	*p*	0.074
Spiritual Comfort	r	−0.124
	*p*	0.113
Environmental Comfort	r	0.309 **
	*p*	<0.001
Motivational Comfort and Hope	r	0.318 **
	*p*	<0.001

Legend: ** *p* < 0.01.

## Data Availability

The data presented in this study are available on request from the corresponding author.

## Public Involvement Statement

There was no public involvement in any aspect of this research.

## Guidelines and Standards Statement

This manuscript was drafted against The Consensus-based Standards for the Selection of Health Measurement Instruments (COSMIN) initiative develops internationally agreed-upon standards and tools to improve the selection and evaluation of health measurement instruments.

## Abbreviations

The following abbreviations are used in this manuscript:

MMSE	Mini-Mental State Examination
PCA	Principal Component Analysis
GCQ	General Comfort Questionnaire
COSMIN	Consensus-based Standards for the Selection of Health Measurement Instruments
CVI	Content Validity Index
KMO	Kaiser–Meyer–Olkin
